# Supplementary material for: Alone in a crowd: effect of a nonfunctional lateral line on expression of the social hormone parathyroid hormone 2
Source: Biol Open. 2022 Oct 18;11(10):bio059432. doi: 10.1242/bio.059432 (PMC9596145; doi:10.1242/bio.059432)
Supplement: Supplementary information [file biolopen-11-059432-s1.pdf]

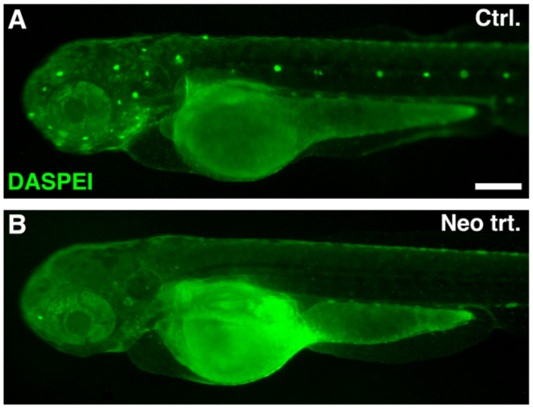

Fig. S1. Vital dye staining of lateral line neuromasts in control and neomycin-treated larvae. Representative 3 dpf larvae from control (A) and neomycin-treated (B) groups stained with DASPEI three hours following a 30-minute treatment with either 0 µM or 50 µM neomycin. Note the near absence of punctate neuromast staining in B, indicating successful ablation of the lateral line hair cells. Scale bar = 200 µm.

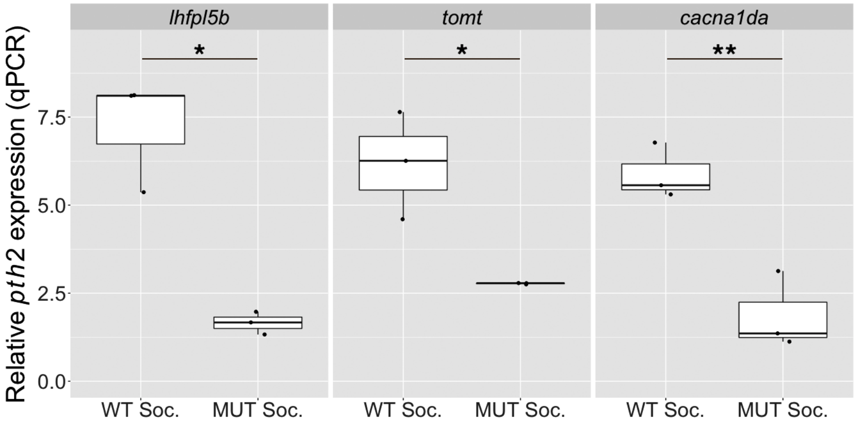

Fig. S2. Boxplots of qPCR results showing *pth2* expression in socially-raised hair cell mutants and wild type siblings at 4 dpf. Ten larval heads were pooled per condition to make one biological replicate, and three biological replicates were used to generate the data. Two technical replicates of the qPCR experiment were conducted per biological replicate and averaged. A one-tailed Welch's t-test was used to assess significance, * = $p < 0.05$, ** = $p < 0.01$.

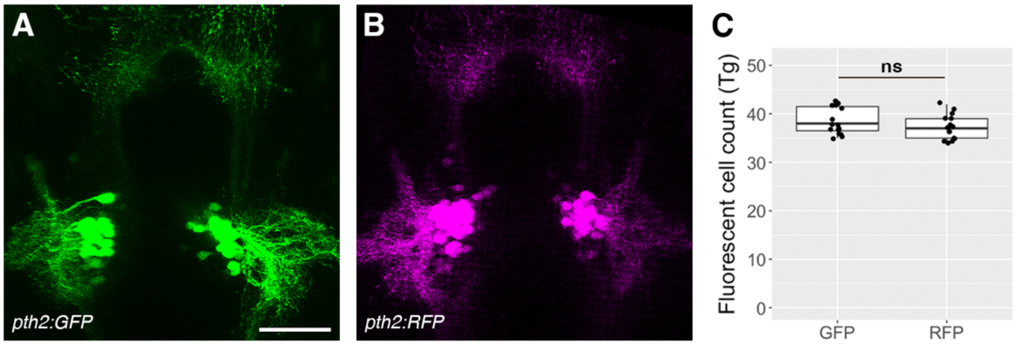

Fig. S3. *Tg(pth2:EGFP)unb2* and *Tg(pth2:TagRFP)unb3* transgenes exhibit similar morphology and number of cells. (A-B) Representative images of socially-reared 4 dpf larva possessing either the A) *Tg(pth2:EGFP)unb2* transgene (green) or B) *Tg(pth2:TagRFP)unb3* transgene (magenta). (C) Boxplot of GFP and RFP cell counts (*n* = 15 larvae per condition). Scale bar is 50 µm. A two-tailed Welch's t-test was used to assess significance, ns = not significant.

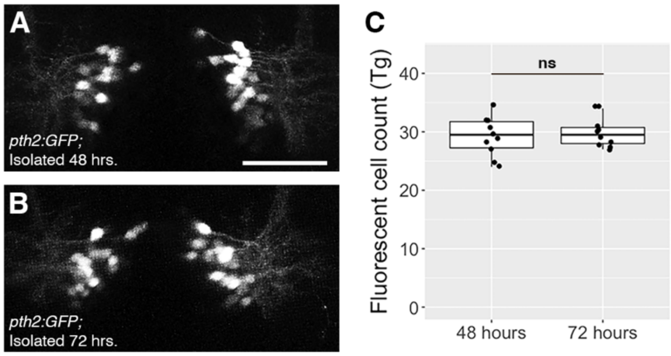

Fig. S4. Increasing the isolation period from 48 to 72 hours does not affect the number of GFP-positive cells in *pth2:GFP* transgenics. (A-B) Representative images of 4 dpf *Tg(pth2:EGFP)unb2* larva raised in isolation for A) 48 hours or B) 72 hours. (C) Boxplots of GFP-positive cell counts for *Tg(pth2:EGFP)* larvae raised in isolation for 48 or 72 hours (*n* = 10 per condition). Scale bar is 50 µm. A two-tailed Welch's t-test was used to assess significance, ns = not significant.

Biology Open • Supplementary information

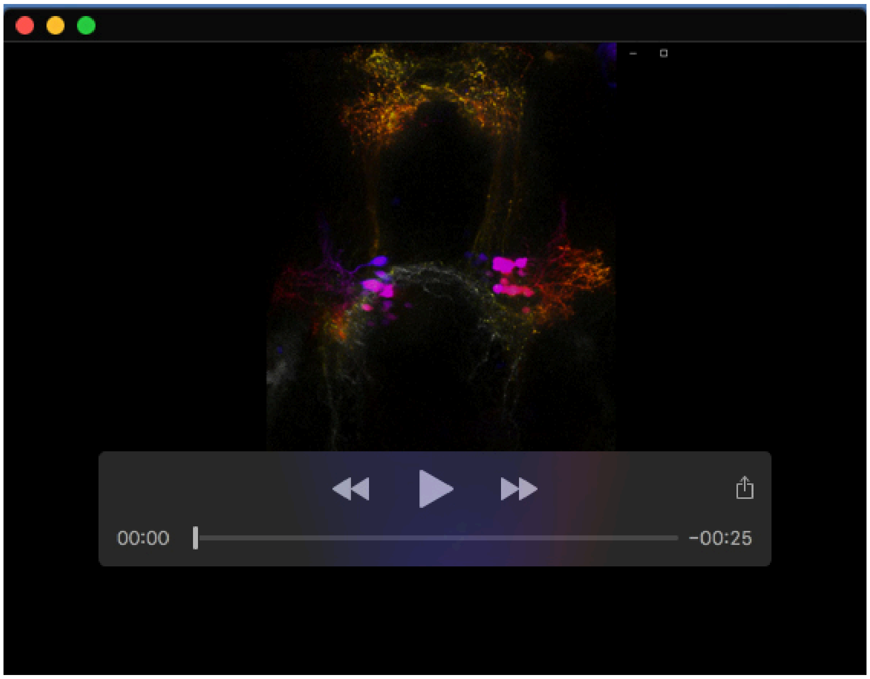

Movie 1. Related to Fig. 4A - Movie showing a 3D representation of cell morphology and neurite projections in a *Tg(pth2:TagRFP)unb3* zebrafish at 5 dpf. Projection depth is 152 µm.
